# Insights Into the Susceptibility of Fungal Infection and STAT3 Genetic Mutations

**DOI:** 10.1111/myc.70200

**Published:** 2026-06-29

**Authors:** Fengming Li, Xiaodong Liu, Yuanyuan Li, Jie Wu, Ningning Dang, Jing Guo

**Affiliations:** ^1^ Department of Dermatology Shandong Provincial Hospital Affiliated to Shandong First Medical University Jinan Shandong Province People's Republic of China

**Keywords:** antifungal immunity, fungal infection, genetic mutations, IL‐17, STAT3

## Abstract

Signal transducer and activator of transcription 3 (STAT3) is a key transcription factor that regulates a spectrum of genes and signalling pathways critical for the antifungal immune response. Mutations in the STAT3 gene confer susceptibility to severe and recurrent fungal infections, predominantly via disruption of interleukin‐17 (IL‐17)‐mediated immunity. This review synthesises current mechanistic insights into STAT3 dysfunction, which impairs the differentiation and effector function of T helper 17 (Th17) cells. This defect results in a profound deficiency of IL‐17 and IL‐22, which are indispensable for orchestrating antifungal defence at mucosal barriers. The subsequent immunopathological features include impaired neutrophil recruitment to fungal invasion sites, compromised epithelial barrier function and failure to eradicate pathogens such as *Candida* spp. and *Aspergillus* spp. Consequently, individuals harbouring STAT3 mutations face a substantially increased risk of disseminated and chronic fungal diseases. Elucidating this STAT3‐dependent signalling pathway is essential for deciphering the pathological basis of fungal susceptibility across diverse clinical settings and informing the development of targeted immunotherapies, including immunomodulatory and genetic therapeutic strategies. Additionally, we performed a systematic review of 135 published cases linking STAT3 mutations to fungal infections, aiming to further characterise the role of STAT3 gene defects in fungal susceptibility.

## 1 Introduction

Fungal infections represent a significant global threat to human health. Pathogenic fungal infections pose serious challenges to public health and individual well‐being. Notably, in individuals with intact immune function, fungal infections are typically restricted to the skin, resulting in superficial dermatological conditions. However, fungal infections can be life‐threatening among immunocompromised populations, including individuals with HIV/AIDS, organ transplant recipients and cancer patients undergoing chemotherapy, potentially leading to severe disease or mortality. Unlike viral infections, there are currently no vaccines or universally effective preventive measures available for fungal pathogens [1]. In recent years, fungal infections caused by environmental and genetic factors have been increasing year by year [2]. Therefore, a comprehensive understanding of the host's antifungal immune mechanisms is essential for developing effective strategies for prevention and treatment.

The signal transducer and activator of transcription (STAT) family is core intracellular signalling mediators that transduce membrane receptor signals to nuclear transcriptional regulation. STAT proteins broadly regulate cell proliferation, differentiation, apoptosis and immune homeostasis and exert indispensable functions in host antifungal immunity [3]. In mammals, the STAT family includes seven highly conserved paralogs: STAT1, STAT2, STAT3, STAT4, STAT5A, STAT5B and STAT6. Functionally, STATs are activated via JAK‐mediated cytoplasmic phosphorylation, followed by dimerisation, nuclear translocation and targeted transcription of downstream genes [4].

STAT proteins share conserved functional domains, among which the SH2 domain mediates dimerisation and the DNA‐binding (DBD) domain governs DNA binding. Notably, most disease‐related loss‐of‐function (LOF) and gain‐of‐function (GOF) mutations cluster within these two domains, indicating shared mutational hotspots across STAT homologues. Nevertheless, individual STAT members are activated by distinct cytokine receptors and control unique transcriptional programs. Accordingly, analogous mutations in conserved domains lead to markedly different disease spectra [5].

Differential pathogenic characteristics of STAT subtype mutations are summarised as follows. STAT1 is a key mediator of interferon signalling. STAT1‐LOF impairs antiviral immunity, whereas STAT1‐GOF disrupts immune homeostasis and predisposes to severe fungal infections [6]. STAT2 deficiency primarily increases viral susceptibility, and STAT2‐GOF drives lethal autoinflammation [7]. As a central regulator of antifungal immunity and tissue repair, transient STAT3 activation orchestrates inflammation, immune defence and wound healing; by contrast, persistent STAT3 activation sustains cell proliferation and anti‐apoptosis [8, 9]. Clinically, STAT3‐LOF causes autosomal dominant hyper‐IgE syndrome (AD‐HIES) with recurrent fungal infections, while STAT3‐GOF leads to multisystem autoimmunity, growth retardation and malignant disorders [5]. STAT4 dominates IL‐12‐driven Th1 development, and its genetic variants confer risks for autoimmune diseases [10]. STAT5 is essential for lymphocyte development; STAT5 deficiency results in combined immunodeficiency and autoimmunity, and STAT5‐GOF is linked to lymphocyte malignancies [11]. As a master regulator of Th2‐mediated allergic inflammation, STAT6‐GOF correlates with severe atopic disorders, whereas pathogenic STAT6‐LOF mutations are rarely reported [5, 12].

Host antifungal defence relies on the crosstalk between innate and adaptive immunity [3]. In innate immune responses, pattern recognition receptors (e.g., Dectin‐1) on neutrophils recognise fungal pathogens, thereby triggering proinflammatory cytokine secretion, ROS production, antimicrobial peptide release and NET formation to eliminate *Candida albicans* and other fungi [13]. In adaptive immunity, Th17 differentiation and downstream cytokines (IL‐17, IL‐22) are central to mucosal antifungal protection [14]. Consistently, the IL‐17 signalling axis is well‐documented to govern mucosal antifungal resistance against cutaneous and mucocutaneous candidiasis [15, 16].

Distinct STAT members participate in antifungal immunity through divergent mechanisms. IL‐6/IL‐23‐dependent STAT3 activation upregulates ROR‐γt, promotes Th17 differentiation and induces IL‐17/IL‐22 production to enhance fungal clearance [14]. STAT1‐GOF mutations represent the major genetic driver of chronic mucocutaneous candidiasis (CMC) [17], which impairs Th1/Th17 responses, reduces IFN‐γ, IL‐17 and IL‐22 secretion and weakens mucosal antifungal defence [18]. In addition, STAT5 phosphorylation facilitates GM‐CSF‐driven neutrophil recruitment to fungal infection sites (e.g., Aspergillus) and initiates effective antifungal reactions [19].

As a key member of the STAT protein family, STAT3 mediates signal transduction from numerous cytokines and growth factors, thereby exerting critical regulatory functions in diverse physiological and pathological processes such as cell proliferation, differentiation, apoptosis and immune modulation [2, 20]. Within the immune system, STAT3 functions as a central mediator of cytokine and growth factor signalling pathways. It plays a critical role in the development, maintenance and functional regulation of immune cells, and is indispensable for the preservation of immune homeostasis [21, 22]. Growing evidence suggests that mutations resulting in STAT3 functional deficiency are strongly linked to increased susceptibility to fungal infections. Impaired STAT3 function undermines the host's antifungal immune defences, thereby facilitating fungal invasion and predisposing individuals to severe clinical outcomes [23, 24]. This review aims to clarify the critical relationship between STAT3 deficiency and increased susceptibility to fungal infections by elucidating the complex mechanisms through which STAT3 regulates antifungal immune responses. We further examine how genetic disruptions in the STAT3 signalling pathway impair host defence against pathogenic fungi. By analysing the molecular basis of antifungal immunity, this review seeks to provide novel insights and identify potential therapeutic strategies to address the growing clinical challenge of invasive fungal diseases.

## 2 STAT3 Introduction

In the context of the Janus kinase (JAK)‐STAT signalling pathway, STAT3 acts as the primary transcription factor, serving as a critical mediator that links extracellular stimuli to intracellular gene expression. It plays an indispensable role in maintaining normal immune function by regulating both innate and adaptive immune responses. Dysregulation of STAT3 leads directly to multiple defects in the innate defence mechanisms of these cells [25]. Thus, a comprehensive understanding of STAT3's structure and function is crucial for elucidating the fundamental mechanisms of cellular signal transduction. Furthermore, it provides a foundational basis for investigating the pathogenesis of fungal infections and developing targeted therapeutic interventions.

### 2.1 Structure of STAT3

**FIGURE 1:**
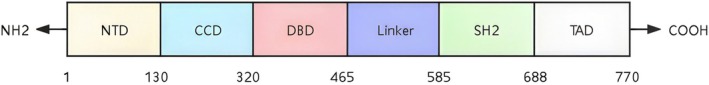
Structural domains of STAT3. STAT3 contains six structural domains: the N‐terminal domain (NTD), the coiled‐coil domain (CCD), the DNA‐binding domain (DBD), the linker domain (LD), the Src homology 2 (SH2) domain and the transcriptional activation domain (TAD).

STAT3 is a member of the STAT protein family, consisting of 770 amino acids with a molecular weight of approximately 88 kDa. The STAT3 gene is located on chromosome 17q21.2 [26]. Four subtypes of STAT3 have been identified: STAT3α, STAT3β, STAT3γ and STAT3δ [27, 28]. As illustrated in Figure 1, STAT3 comprises six structural domains: the N‐terminal domain (NTD), the coiled‐coil domain (CCD), the DNA‐binding domain (DBD), the linker domain (LD), the Src homology 2 (SH2) domain and the transcriptional activation domain (TAD). The NTD contributes to multiple functions, such as facilitating nuclear translocation of STAT3 dimers and mediating cooperative DNA binding. The CCD is critical for recruiting STAT3 to membrane‐associated receptors. The DBD allows STAT3 to specifically recognise and bind target DNA sequences. The LD links the DBD and SH2 domains and is vital for transcriptional activation. The SH2 domain is essential for receptor binding, phosphorylation and STAT3 dimerisation. The TAD contains two key phosphorylation residues: tyrosine 705 and serine 727. The coiled‐coil domain, located in the N‐terminal region, facilitates interactions between STAT3 proteins and the formation of complexes with other proteins. Adjacent to this region lies the DNA‐binding domain, which allows STAT3 to bind specific DNA sequences, forming the structural basis for its role in transcriptional regulation. The C‐terminal region contains the transcriptional activation domain, which includes multiple phosphorylation sites. Upon stimulation by specific cytokines or growth factors, tyrosine residues within STAT3 are phosphorylated, activating the TAD. This activation leads to the recruitment of transcriptional cofactors, initiation and elongation of gene transcription, and ultimately regulation of downstream target gene expression [29, 30].

### 2.2 Regulatory Mechanisms of the STAT3 Signalling Pathway

The regulatory mechanisms governing the STAT3 signalling pathway are highly complex and involve interactions across multiple levels and molecules. Understanding these mechanisms is crucial for clarifying cellular physiological functions and the pathogenesis of associated diseases. The JAK–STAT pathway represents a pivotal conduit for mediating cytokine signalling. This pathway is subject to the influence of multiple factors, including cytokine diversity, receptor expression profiles, JAK/STAT subtype specificity and the actions of negative feedback regulators such as SOCS, thereby controlling biological processes including cell proliferation, differentiation, immune responses and metabolism [5, 31]. STAT3 is a component of the JAK/STAT signalling pathway and is one of the most intricate transcriptional regulators involved in diverse physiological processes. It mediates the transmission of signals from the cell membrane to the nucleus, thereby coordinating cellular activities both inside and outside the cell [32].

**FIGURE 2:**
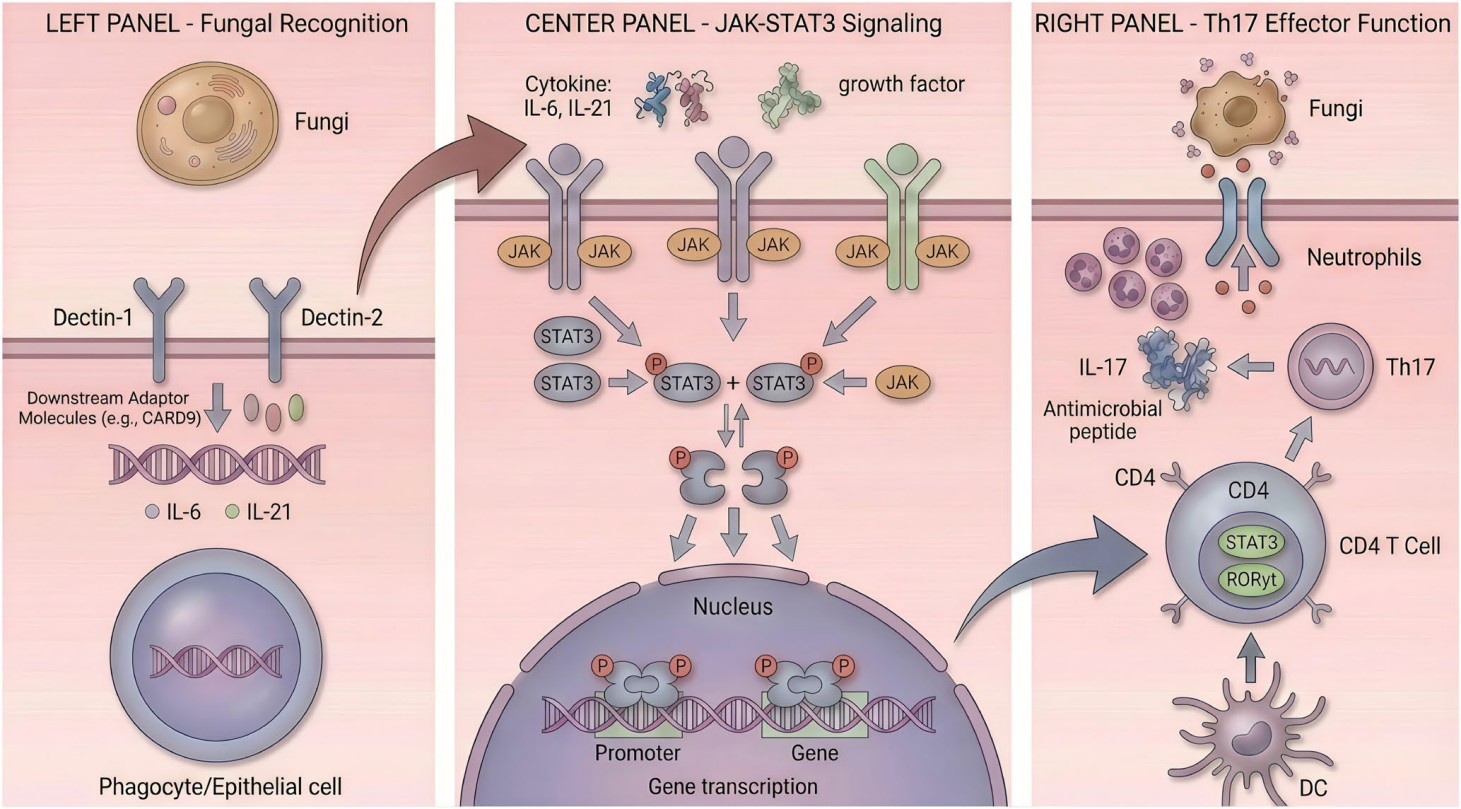
STAT3 activation. Upon ligation to cell‐surface pathogen recognition receptors (e.g., Dectin‐1 and Dectin‐2), downstream adaptor molecules including CARD9 drive the production of proinflammatory cytokines (IL‐6, IL‐21) in myeloid and epithelial cells through the NF‐κB and AP‐1 signalling pathways. These cytokines and growth factors bind to corresponding receptors on the cell membrane, causing receptor dimerisation. This subsequently activates JAK kinases, which in turn phosphorylate STAT3. Phosphorylated STAT3 dimerises and translocates to the nucleus, where it recognises specific DNA sequences to initiate transcription of downstream target genes. Concurrently, activated STAT3 binds to RORγt protein to form a transcription complex. This complex further promotes Th17 cell maturation and IL‐17 secretion. IL‐17 effectively recruits neutrophils to the infection site to eliminate pathogens.

In its inactive state, STAT3 exists as monomers in the cytoplasm [33]. In humans, the mechanism through which STAT3 mediates cellular effects involves three sequential steps: phosphorylation by specific kinases, phosphorylation‐induced dimerisation and transcriptional activation by the resulting phosphorylated dimers [34] (Figure 2). STAT3 can be activated by a variety of cytokines and growth factors, including those that signal through JAKs and tyrosine kinases. When cells encounter stimulation from pathogenic microorganisms or other stimuli, the classical JAK–STAT3 signalling pathway is initiated. Ligands bind to cell surface receptors, inducing receptor dimerisation. These dimerised receptors then recruit and activate JAK kinases in the cytoplasm. Activated JAKs phosphorylate tyrosine 705 and serine 727 on STAT3, leading to its activation [34, 35]. Following activation, STAT3 dimerises through its SH2 domain, translocates to the nucleus and initiates transcriptional activation of target genes [35, 36, 37, 38].

In addition to direct phosphorylation, STAT3 activity can also be regulated by various post‐translational modifications, such as acetylation, methylation and mono‐ubiquitination. These modifications indirectly influence STAT3 phosphorylation and play an important role in fine‐tuning its transcriptional activity [39]. For instance, the acetylation of multiple lysine residues in the NH2‐terminal domain and SH2 domain of STAT3 (predominantly catalysed by CBP/p300) has been demonstrated to enhance its capacity for transcriptional activation. This enhancement is closely associated with increased STAT3 dimer stability, elevated phosphorylation levels at the Tyr705 site, enhanced nuclear translocation efficiency and high levels of histone acetylation in the promoter regions of target genes [40]. Conversely, when acted upon by deacetylases such as HDAC1‐3, SIRT1, or LOXL3, the transcriptional activity of STAT3 is inhibited [41, 42, 43]. Consequently, the dynamic equilibrium between acetylation and deacetylation represents a pivotal mechanism governing STAT3 activation and its downstream cellular functions [44, 45]. In addition, methylation and ubiquitin‐like modifications have emerged as critical regulatory pathways. SMYD2‐dependent methylation promotes hyperphosphorylation of STAT3, whereas EZH2‐ and SET9‐mediated dimethylation inhibit the DNA‐binding ability of STAT3 dimers. Moreover, SUMO2/3 modification of STAT3 at lysine 451 enhances its interaction with the nuclear phosphatase TC45, thereby sequestering phosphorylated STAT3 in the nucleus. In contrast, SENP3‐mediated de‐sumoylation leads to aberrantly elevated phosphorylation levels of STAT3 [46].

### 2.3 STAT3 Mutation

Germline mutations in STAT3 predominantly affect the SH2, DBD and TAD. The functional consequences of these mutations determine their classification as either LOF or GOF variants [47]. STAT3‐LOF mutations are primarily associated with increased susceptibility to fungal infections due to impaired immune responses, whereas STAT3‐GOF mutations are more commonly linked to autoimmune disorders, malignancies and other chronic pathological conditions [48].

#### 2.3.1 STAT3 Loss‐Of‐Function Mutations

Patients with STAT3‐LOF mutations exhibit impaired STAT3 protein expression, leading to disrupted IL‐17 and IL‐22 signalling pathways. This defect severely compromises Th17 cell differentiation and markedly reduces IL‐17 production, resulting in widespread abnormalities in the host innate immune system. Given the critical role of the Th17/IL‐17 axis in defending against fungal and bacterial pathogens, STAT3‐LOF mutations significantly impair antifungal immunity. Furthermore, these patients display deficiencies in B memory cells and multiple T cell subsets, characterised by increased numbers of naïve CD8^+^ T cells and reduced proportions of effector, central memory and terminally differentiated memory cytotoxic T cells. These immunological alterations are associated with diminished responsiveness to cytokine stimulation and impaired anti‐fungal activity [49, 50].

The association between STAT3‐LOF mutations and fungal infections is primarily mediated by the clinical phenotype of hyper‐IgE syndrome (HIES). To date, STAT3 remains the only known human gene in which pathogenic variants can lead to autosomal dominant HIES (AD‐HIES), a condition now commonly termed STAT3‐HIES. This congenital immunodeficiency is characterised by a classic triad of symptoms: elevated serum IgE levels, atopic dermatitis and recurrent skin and pulmonary infections [51, 52, 53]. Fungal infections are prevalent in patients with STAT3‐HIES. The most commonly observed manifestations include cutaneous, oropharyngeal and vaginal candidiasis, as well as cystic pulmonary lesions. Opportunistic infections such as Pneumocystis pneumonia, disseminated histoplasmosis and secondary alveolar infections caused by *Aspergillus* or *Streptomyces* species may also occur [52, 54].

#### 2.3.2 STAT 3 Gain‐Of‐Function Mutations

STAT3‐GOF mutations are associated with susceptibility to fungal infections [48, 55]. Mutations causing STAT3‐LOF predominantly occur in the DNA DBD and SH2 domain. STAT3‐GOF mutations are widely distributed across various protein domains, with the majority occurring in the DBD and CCD. Most of these mutations are missense mutations [56, 57]. Jennifer et al. noted that a hallmark feature of STAT3 gain‐of‐function syndrome is autoimmune cytopenia [58]. Approximately 25% of patients develop secondary fungal infections, with significantly elevated risk particularly in those with CD3^+^ or CD4^+^ T lymphopenia [58].

However, the precise pathogenic mechanisms remain incompletely elucidated. Further analysis of peripheral blood immune cells from patients by Jennifer et al. revealed that only a minority of STAT3‐GOF patients exhibited elevated Th17 cell proportions, while some individuals instead showed CD8^+^ T cell expansion, suggesting immune response heterogeneity [58]. To simulate human immunopathological states, Kelsey et al. generated a STAT3‐GOF mouse model harbouring the pathogenic p.G421R mutation [59]. They observed that these mice spontaneously developed or were induced by imiquimod to exhibit skin inflammation, with the phenotype correlated to enhanced local Th17 responses. This demonstrates the critical role of Th17 cells in STAT3‐GOF associated immune dysregulation [59]. Given the crucial role of the Th17/IL‐17 axis in antifungal immunity, even though the aforementioned studies did not directly uncover a molecular link between STAT3‐GOF and fungal infection, the abnormal Th17 response induced by this mutation may indirectly undermine the body's defence against fungi, thus increasing the susceptibility to infection. STAT3‐GOF mutations may exhibit complex and indirect relationships with fungal infections by disrupting the balance of T lymphocyte subsets.

## 3 Immune Regulation of STAT3

STAT3 plays a pivotal and multifaceted role in the immune system, being essential for maintaining immune homeostasis and systemic stability. The immune response can broadly be categorised into two main types: innate immunity and adaptive immunity. The innate immune system is mediated by diverse populations of innate immune cells, including myeloid cells, natural killer (NK) cells and innate lymphoid cells, as well as evolutionarily conserved humoral components such as defensins and complement proteins [60]. This system responds rapidly to pathogen invasion but lacks antigen specificity. In contrast, adaptive immunity is present in jawed vertebrates, is mediated by members of the immunoglobulin superfamily and cellular components such as B lymphocytes and T lymphocytes. Although it responds more slowly upon initial exposure to pathogens, it exhibits high specificity and is capable of generating long‐lasting immune memory [61]. Upon fungal infection, the innate immune system serves as the first line of defence, rapidly activating within minutes to hours to control and eliminate the pathogen. However, when the pathogen load is high or the fungus exhibits high virulence, innate immune mechanisms may be insufficient to fully clear the infection. In such cases, lymphocytes and adaptive immune responses are recruited to mount a specific and targeted response against the invading fungi [62].

Upon fungal invasion, pathogens initially interact with endothelial cells (ECs), eliciting an innate immune response. STAT3 activation in ECs promotes the synthesis of granulocyte colony‐stimulating factor (G‐CSF), the principal growth factor regulating neutrophil production, through modulation of key steps in emergency granulopoiesis. This process facilitates the rapid mobilisation of mature neutrophils from bone marrow reservoirs and concurrently initiates de novo granulocyte generation, ensuring a sustained supply of neutrophils to effectively control the infection [63, 64]. However, STAT3 also serves to restrain neutrophil production and modulate the inflammatory response. A critical negative regulatory mechanism involves the STAT3‐dependent induction of suppressor of cytokine signalling 3 (SOCS3), which inhibits the activity of the granulocyte colony‐stimulating factor receptor (G‐CSFR) and other cytokine receptor families [65]. Upon cytokine stimulation, STAT3 rapidly induces SOCS3 gene transcription. The newly synthesised SOCS3 protein accumulates in the cytoplasm and concurrently engages the receptor/JAK complex via two distinct interfaces, thereby suppressing receptor‐mediated signal transduction. Furthermore, the kinase inhibitory region located near the N‐terminus of SOCS3 directly interacts with receptor‐associated JAK proteins, effectively blocking their ability to phosphorylate downstream substrates [66, 67]. As a result, G‐CSF binding to its receptor is restricted, leading to diminished neutrophil production. Thus, STAT3 not only coordinates emergency granulopoiesis and neutrophil migration but also limits the duration of these responses to prevent excessive or tissue‐damaging granulocyte accumulation.

STAT3 plays a critical role in the development of plasmacytoid dendritic cells (DCs) and conventional dendritic cell lineages. Fms‐like tyrosine kinase 3 ligand (Flt3L), an exogenous growth factor, serves as the primary regulator of dendritic cell expansion in vivo. STAT3 is essential for optimal Flt3L‐mediated dendritic cell development, as it upregulates Flt3 expression, facilitates dendritic cell differentiation and sustains dendritic cell immunological function [68, 69, 70]. Moreover, in mature phagocytes, STAT3 serves as a critical negative regulator of Toll‐like receptor (TLR)‐mediated signalling, particularly for TLR4, TLR2 and TLR9. For instance, macrophages, neutrophils and DCs lacking STAT3 produce abnormally high levels of pro‐inflammatory cytokines such as TNF‐α, IL‐6, IL‐12 and IFN‐γ upon TLR4 activation. Although these STAT3‐deficient cells also exhibit increased IL‐10 production, they fail to respond to this key anti‐inflammatory cytokine, thereby predisposing to the development of inflammatory diseases [71].

When the pathogen load is high or the invading pathogens exhibit high virulence, and the innate immune system fails to effectively control the infection, the adaptive immune system is activated. This response is primarily mediated by B lymphocytes, CD4^+^ T lymphocytes and CD8^+^ T lymphocytes [65]. In B cells, STAT3 is critical for the functional activity of various B cell subsets, the progression of B cell progenitors into mature precursors, and the survival of these developing cells [72]. Owing to its responsiveness to IL‐35 receptor signalling, STAT3 may drive the induction of IL‐10 and IL‐35 secretion in IL‐35–induced regulatory B cells (Bregs), which play a key role in suppressing excessive immune responses, modulating autoimmune reactions and maintaining immune homeostasis [73]. In T lymphocytes, STAT3 is essential for the differentiation of naïve CD4^+^ T cells into IL‐17‐producing Th17 cells. Specifically, STAT3 activates the expression of retinoic acid RORγt and RORα, lineage‐defining transcription factors required for Th17 development [74, 75]. These transcription factors are indispensable for the generation of Th17 cells, which are vital for host defence against both extracellular and intracellular fungal pathogens. Furthermore, STAT3 plays a central role in the formation of long‐lived memory T cells. Upon re‐encounter with the same pathogen, these memory T cells can be rapidly reactivated, enabling a faster and more robust secondary immune response. Through the coordination of multiple signalling pathways, STAT3 ensures the proper establishment and sustained function of immune memory [76]. In summary, STAT3 is fundamentally involved in regulating major components of the immune system. Its physiological activity is crucial for effective host defence against pathogenic invasion and for the maintenance of immune balance.

### 3.1 The Role of STAT3 in Antifungal Immunity

STAT3 plays a multifaceted and critical role in antifungal immunity. Upon fungal invasion, the innate immune system is rapidly activated. Antigen‐presenting cells recognise fungal antigens and present them to dendritic cells and myeloid cells of the T lymphocyte lineage, initiating a cascade of immune activation. These cells subsequently secrete inflammatory cytokines that stimulate the STAT3 signalling pathway, thereby promoting the recruitment and functional activation of neutrophils, natural killer cells and other effector immune cells to eliminate invading fungi [77]. When the innate immune response is insufficient to eliminate fungal pathogens, the adaptive immune system is activated. Adaptive immune cytokines enhance neutrophil responses to fungal infection. Key adaptive immune cells include B lymphocytes, helper T cells (Th), regulatory T cells (Tregs) and cytotoxic T cells, which work together to initiate inflammatory responses and induce the formation of neutrophil extracellular traps (NETs) [78]. Th17 cells are potent inducers of neutrophils and directly stimulate NET formation [79]. Neutrophils, in turn, amplify this response through a feedforward loop: NETs promote STAT3 phosphorylation via their protein components, enhancing the expression of the Th17 lineage‐specific transcription factor RORγt and thereby promoting Th17 cell differentiation and further NET formation [80, 81]. Neutrophils eliminate fungi primarily through phagocytosis, driven by oxidative bursts mediated by NADPH oxidase activity [82].

#### 3.1.1 The Role of Th17/IL‐17 Axis in Antifungal Immunity

The STAT3 signalling pathway is essential for the differentiation of Th17 cells. Patients with STAT3 deficiency exhibit a marked reduction in Th17 cell numbers and impaired IL‐17 secretion [83, 84]. IL‐17 is primarily secreted by various immune cells, including Th17 cells, CD8^+^ T cells, γδ T cells, NK T cells and innate lymphoid cells, in response to infection and inflammation. It serves as a key effector molecule in mucosal immunity against *Candida* [85]. Extensive research has elucidated the central role played by Th17 cells and their effector factor IL‐17 in the pathogenesis of autoimmune diseases [86]. The IL‐17 cytokine family comprises six related members: IL‐17A, IL‐17B, IL‐17C, IL‐17D, IL‐17E and IL‐17F [86]. These members share a highly similar C‐terminal structure, characterised by 20%–30% amino acid sequence identity and four strictly conserved cysteine residues. The core immunological function of IL‐17 is to coordinate the host's anti‐infective defences. On the one hand, IL‐17 has been demonstrated to induce the production of pro‐inflammatory cytokines, chemokines and other mediators, thereby effectively recruiting neutrophils to the site of infection to eliminate pathogens. Conversely, it has been observed to stimulate cells in the skin and mucosal surfaces to produce antimicrobial peptides (AMPs), including defensins. This provides the host with direct and rapid extracellular fungal defence [87, 88].

IL‐17A is the most extensively characterised member of this family. Encoded by a gene on human chromosome 6p12, the IL‐17A protein consists of 150 amino acids, has a molecular weight of approximately 17 kDa, and contains three potential N‐linked glycosylation sites [89].

In healthy individuals, IL‐17A and related cytokines are crucial for innate immunity against extracellular fungi. They function by protecting the epithelial and mucosal barriers of the skin, colon and respiratory tract, thereby maintaining effective defence at these critical interfaces [90, 91]. Th17 cells secrete IL‐17 and stimulate the production of antimicrobial peptides, thereby contributing to the adaptive immune response against fungal pathogens [52]. Upon fungal invasion, pattern recognition receptors, including Dectin‐1 and Toll‐like receptors (TLRs), recognise fungal components such as spores and hyphae, triggering innate immune activation and initiating the differentiation of Th1 and Th17 cells [92, 93]. DCs process and present fungal antigens to naïve T cells, driving their differentiation into Th1 and Th17 lineages, which bridges innate and adaptive immunity. IL‐6 signalling activates STAT3, which in turn induces the expression of retinoic acid RORγt, a master transcription factor essential for Th17 cell development and IL‐17 production. Following differentiation, Th17 cells enhance host defence by recruiting and activating neutrophils at the site of infection, promoting the release of pro‐inflammatory cytokines and chemokines, and secreting IL‐22 to stimulate epithelial cells to produce antimicrobial peptides [14].

#### 3.1.2 STAT3 and Th17/IL‐17 Axis

Given that STAT3 serves as the primary mediator of the IL‐6 signalling pathway and that IL‐6 is a critical driver of Th17 cell differentiation, the pivotal role of STAT3 in this process is unequivocally established [94, 95]. It is well established that STAT3 serves as a critical upstream regulator of IL‐17 expression, primarily through its regulation of the transcription factor RORγt. Upon IL‐23 binding to its receptor, STAT3 is phosphorylated and activated. The activated STAT3 subsequently dimerises and translocates into the nucleus, where it directly binds to the promoter region of the RORγt gene to enhance its transcription. Furthermore, STAT3 interacts with the RORγt protein to form a functional transcriptional complex, thereby amplifying IL‐17 production. This complex further binds to the promoter region of the IL‐17 gene, thereby driving its transcriptional activation and facilitating Th17 cell differentiation. Moreover, STAT3 promotes Th17 cell survival by directly inducing anti‐apoptotic signalling pathways. As a result, STAT3 and RORγt establish a positive feedback loop that sustains elevated IL‐17 production and stabilises the functional identity of Th17 cells [96]. It has been demonstrated that phosphorylation of STAT3, for example at the Tyr705 site, directly suppresses IL‐17 expression, thereby indicating that impaired STAT3 phosphorylation contributes to reduced immune defence. In animal models, STAT3 deficiency has been shown to impair the clearance of *Candida* from oral, oesophageal and intestinal mucosal surfaces [97]. Studies on functional redundancy in the IL‐23/IL‐17 axis reveal that mutations in the IL‐23 receptor similarly disrupt IL‐17 secretion and increase susceptibility to mucosal fungal infections [98, 99]. Furthermore, STAT3 plays a critical role in IL‐6 trans‐signalling‐induced Th17 differentiation by regulating the SIRT2/LDHA axis, and its dysregulation further compromises mucosal barrier integrity [100].

In the mouse cGVHD model, inhibition of STAT3 phosphorylation was found to reduce the expression of IRF4 and RORγt in splenocytes. RORγt, a splice variant of the nuclear receptor RORγ that is selectively expressed in T cells—particularly in in vitro‐differentiated Th17 cells—is a key transcription factor for Th17 lineage commitment. Furthermore, IRF4 is essential for IL‐17 regulation in murine autoimmunity. Thus, the downregulation of these two factors upon STAT3 inhibition underscores STAT3's critical role in the Th17 pathway [101]. This finding indicates that STAT3 functions as the predominant transcriptional activator in Th17 cells. It plays a pivotal role in regulating Th17 cell development and is indispensable for in vivo Th17 differentiation.

Although STAT3 deficient patients predominantly exhibit Th17 cell dysfunction, the aberrantly enhanced type 1 immune response also contributes to their increased susceptibility to fungal infections. Evidence from studies indicates that in STAT3‐deficient mouse models, excessive production of IFN‐γ can lead to epithelial barrier impairment via STAT1‐dependent pathways, thereby compromising mucosal integrity and facilitating invasion by fungal pathogens such as *Candida* [102]. Concurrently, macrophage functionality is impaired; in the absence of IFN‐γ‐JAK–STAT signalling, their fungicidal capacity is significantly reduced, a process mediated by diminished phagocytic activity and dysregulation of inflammatory cytokine balance [102, 103]. Another study has shown that in patients with STAT3 deficiency, PBMCs display impaired Th17 responses following fungal stimulation, as well as aberrant Th1 responses characterised by dysregulated IFN‐γ production [104]. This finding indicates that STAT3 plays a dual regulatory role in modulating Th1 and Th17 immune responses. Further studies have shown that patients' susceptibility to fungal infections is not solely attributable to the deficiency of Th17 cells. Rather, heightened IFN‐γ signalling actively impairs residual Th17 function through the JAK–STAT1 pathway, leading to a condition of ‘functional immunodeficiency’.

Furthermore, STAT3 dysfunction has been shown to trigger aberrant inhibitory signalling through immune receptor tyrosine‐based activation motif (ITAM)‐associated pathways. Studies have demonstrated that heightened signalling via monocyte ITAM receptors (e.g., Dectin‐1 and Dectin‐2) commonly co‐occurs with Th17 cell impairment in patients harbouring STAT1‐GOF mutations [103, 105]. Although Dectin‐1/2 deficiency has been shown to impair host defence against *Cryptococcus neoformans* , the protective mechanism involved does not seem to rely on IL‐17. This raises the possibility that STAT3 may regulate innate immune recognition by modulating the NFAT5/NonO axis downstream of ITAM‐coupled receptors [106, 107]. Furthermore, upon activation, SHP2 phosphatase binds to the IL‐6 receptor, disrupting the interaction between STAT3 and the receptor. This disruption has been shown to inhibit Th17 cell differentiation and increase susceptibility to fungal infections [108].

STAT3 plays a critical role in maintaining cellular redox homeostasis through the regulation of metabolic processes and stress responses. Functional deficiency of STAT3 may impair antioxidant defences, thereby amplifying oxidative stress‐induced damage. Acetylation of STAT3, particularly via SIRT2‐dependent mechanisms, has been shown to modulate lactate dehydrogenase A (LDHA) activity, influencing glycolytic flux and intracellular redox balance [100]. Clinical evidence indicates that patients with STAT3 deficiency are susceptible to chronic pulmonary *Aspergillus* infections and frequently develop tissue‐invasive lesions, a tendency likely driven by sustained accumulation of oxidative damage in affected tissues [47, 109]. Moreover, although direct experimental validation is still limited, STAT3 may contribute to genomic stability in immune cells within inflammatory environments through transcriptional regulation of DNA repair‐related genes. Impaired STAT3 function could thus exacerbate DNA damage in immune cells during fungal infections, potentially compromising their viability and functional integrity.

## 4 Fungal Infections in Patients with STAT3 Mutations

The present study systematically evaluates the clinical association between STAT3 gene mutations and fungal infections. A comprehensive search of the PubMed database was conducted to identify relevant English‐language articles published since 2015. The search strategy incorporated a combination of key terms: ‘STAT3 deficiency’ OR ‘STAT3 mutation’ OR ‘STAT3’ AND ‘*Candida*’ OR ‘*Aspergillus*’ OR ‘*Talaromyces marneffei* (TM)’ OR ‘fungi’. Following an extensive review of the retrieved literature, the following inclusion and exclusion criteria were applied: (1) patients with confirmed HIV infection were excluded; (2) studies not directly related to STAT3 gene mutations were excluded; and (3) reports lacking definitive evidence of fungal infection were excluded. The final analysis included data from 135 patients with documented STAT3 gene mutations and concurrent fungal infections, distributed across multiple regions including China, Europe, India, the United States and other countries. The spectrum of fungal infections comprised 85 cases of *Candida* infection [110, 111, 112, 113, 114], 25 cases of *Aspergillus* infection [24, 47, 115, 116, 117, 118, 119], 20 cases of *TM* infection [120, 121, 122, 123, 124, 125, 126, 127] and 5 cases of *Fusarium* infection [83, 128] (see Table 1 for details). In addition, for the 84 patients whose samples contained clear mutation information (including one patient with two mutations, making a total of 85 mutation types), we calculated the frequency of different gene mutations and presented their distribution as percentages (see Table 2 for details). Subsequent sections will explore the underlying relationship between STAT3 mutations and susceptibility to fungal infections through an analysis of representative infection types.

**TABLE 1:** Fungal infections in patients with STAT3 mutations.

Fungal infections types	Number of cases	Mutations	Gender (M/F)	Age (y/m/d)	Region	Ref.
*Candida* (85 cases)	33	Not reported	Not reported	Median 34.6 y	Europe	[110]
	15	Not reported	Not reported	Median 9.8 y	India	[111]
	21	c.1594A>C (p.K531Q)	Not reported	16 y	Iran	[112]
		c.995G>A (p.H332Y)		31 y	Iran	
		c.1144C>T (p.R382W)		1 y	Iran	
		c.1145G>A (p.R382Q)		2 y	Peru	
		c.1909G>A (p.V637M)		9 y	Iran	
		c.1850G>A (p.G617E)		2 y	Morocco	
		c.2144 + 1G>A (p.?)		8 y	Morocco	
		c.1971delT (p.Y657*)		19 y	Iran	
		c.2131A>T (p.I711F)		4 y	Germany	
		c.1387_1389del (p.V462del)		12 y	Turkey	
		c.1591A>G (p.K531E)		5 y	Great Britain	
		c.1395_1397del (p.N466del)		4 y	Colombia	
		c.2125A>G (p.K709E)		3 y	Germany	
		c.1144C>G (p.R382G)		1 y	Turkey	
		c.1723T>G (p.Y575D)		36 y	Germany	
		c.1711C>T (p.L571F); c.1110‐2A>G (p.?)		1 y	Great Britain	
		c.1909G>A (p.V637M)		1 y	Egypt	
		c.1909G>A (p.V637M)		1 y	Macedonia	
		c.1708G>A (p.D570N)		1 y	Great Britain	
		c.1680_1682del (p.F561del)		Not reported	Great Britain	
		c.1708G>A (p.D570N)		29 y	Great Britain	
	14	c.1859C>G (p.T620S)	M	3 m	China	[113]
		c.1909G>A (p.V637M)	F	6 m		
		c.1145G>A (p.R382Q)	M	3 d		
		c.1909G>A (p.V637M)	F	3 d		
		c.1825A>G (p.R609G)	F	3 d		
		c.1909G>A (p.V637M)	F	1 d		
		c.1907C>T (p.S636F)	M	2 d		
		c.1144C>T (p.R382W)	M	40 d		
		c.1145G>A (p.R382Q)	M	3 m		
		c.1144C>T (p.R382W)	M	10 d		
		g.76304T>G (p.C712G)	M	5 m		
		c.1294G>A (p.V432M)	F	1 d		
		c.1144C>T (p.R382W)	M	2 m		
		c.2113T>C (p.Y705H)	M	3 d		
	2	c.1166C>T (p.T389I)	M	3 m	Israel	[114]
		c.1166C>T (p.T389I)	F	3 m		
*Aspergillus* (25 cases)	1	c.109C>T (p.W37*)	F	22 y	Not reported	[47]
	6	c.1144C>T (p.R382W)	Not reported	Not reported	Not reported	[24]
		c.1144C>T (p.R382W)				
		c.1144C>T (p.R382W)				
		c.1144C>T (p.R382W)				
		c.1680_1682del (p.F561del)				
		c.1909G>A (p.V637M)				
	13	c.1387_1389del (p.V462del)	M	10 y	Not reported	[115]
		c.1909G>A (p.V637M)	F	11 y		
		c.1145G>A (p.R382Q)	F	13 y		
		c.1144C>T (p.R382W)	F	10 y		
		c.1909G>A (p.V637M)	M	28 y		
		c.1144C>T (p.R382W)	M	12 y		
		c.1909G>A (p.V637M)	F	10 y		
		c.1680_1682del (p.F561del)	F	21 y		
		c.1144C>T (p.R382W)	M	10 y		
		c.1145G>A (p.R382Q)	M	33 y		
		c.1282‐89C>T (p.?)	F	26 y		
		c.1282‐89C>T (p.?)	M	20 y		
		c.1971C>G (p.Y657C)	M	35 y		
	2	c.1144C>T (p.R382W)	Not reported	12 y	France	[116]
		c.1282‐89C>T (p.?)		20 y		
	1	c.1294G>T (p.V432L)	F	28 y	China	[117]
	1	c.1909G>A (p.V637M)	F	1.5 y	China	[118]
	1	c.*1671C>T (p.=)	M	37 y	United Nations	[119]
*TM* (20 cases)	1	Not reported	M	Not reported	China	[120]
	1	c.1394C>T (p.S465F)	F	8 m	China	[121]
	7	c.115G>A (p.E39K)	Not reported	Not reported	China	[122]
		c.1679_1681del (p.T560del)				
		c.1593A>T (p. K531N)				
		c.1593A>T (p.K531N)				
		c.92G>A (p.R31Q)				
		c.1121A>G (p.D374G)				
		c.1673G>A (p.G558D)				
	6	c.1121A>G (p.D374G)	F	1 y	China	[123]
		Not reported	M	10 y		
		c.1593A>T (p.K531N)	M	13 y		
		c.1673G>A (p.G558D)	M	3 y		
		c.92G>A (p.R31Q)	M	34 y		
		Not reported	F	2 y		
	1	c.1673G>A (p.G558D)	M	3 y	China	[124]
	1	c.1909G>A (p.V637M)	M	18 y	China	[125]
	2	c.1679_1681del (p.T560del)	Not reported	Not reported	China	[126]
		c.1593A>T (p.K531N)				
	1	c.92G>A (p.R31Q)	F	33 y	China	[127]
*Fusarium* (5 cases)	4	c.1144C>T (p.R382W)	M	Not reported	France	[83]
		c.1144C>T (p.R382W)	M	26 y		
		c.2003C>T (p.T668M)	M	11 y		
		c.1145G>A (p.R382Q)	F	4 y		
	1	c.1027G>T (p.V343F)	F	2 y	China	[128]
Total	135 cases					

*Note:* This table categorises cases by fungal infection type and summarises the distribution of STAT3 mutations across different fungal pathogens. Data are aggregated from the cited references. Ages are presented as years (y), months (m), or days (d) as appropriate. ‘Not reported’ indicates missing data in the original study. The mean age for the *Candida* cohort is 34.6 years, ages for other cohorts are listed as reported in the references.

Abbreviations: F, female; M, male; p.?, undetermined amino acid variation; p.=, synonymous variant; TM, *Talaromyces marneffei*.

**TABLE 2:** Distribution of STAT3 gene mutation frequencies in this study.

Mutations	Number of cases	Percentage	Main types of infection
c.1144C>T (p.R382W)	14	16.47	*Candida, Aspergillus; Fusarium*
c.1909G>A (p.V637M)	12	14.12	*Candida, Aspergillus, TM*
c.1145G>A (p.R382Q)	6	7.06	*Candida, Aspergillus; Fusarium*
c.1593A>T (p. K531N)	4	4.71	*TM*
c.1680_1682del (p.F561del)	3	3.53	*Candida, Aspergillus*
c.1282‐89C>T (p.?)	3	3.53	*Aspergillus*
c.92G>A (p.R31Q)	3	3.53	*TM*
c.1673G>A (p.G558D)	3	3.53	*TM*
c.1708G>A (p.D570N)	2	2.35	*Candida*
c.1166C>T (p.T389I)	2	2.35	*Candida*
c.1387_1389del (p.V462del)	2	2.35	*Candida, Aspergillus*
c.1679_1681del (p.T560del)	2	2.35	*TM*
c.1121A>G (p.D374G)	2	2.35	*TM*
Other unique mutations	27	31.77	*Candida, Aspergillus, TM, Fusarium*
Total	85	100	

*Note:* Percentage calculations are based solely on the 84 patients in this table for whom mutation information is recorded (including one patient with two mutations, making a total of 85 mutations). Cases with unreported mutations were not included in the statistics. Of these, 27 mutation types in the ‘Other unique mutations’ section were reported in only one case each. These include: c.1594A>C (p.K531Q); c.995G>A (p.H332Y); c.1850G>A (p.G617E); c.2144+1G>A (p.?); c.1971delT (p.Y657*); c.2131A>T (p.I711F); c.1591A>G (p.K531E); c.1395_1397del (p.N466del); c.2125A>G (p.K709E); c.1144C>G (p.R382G); c.1723T>G (p.Y575D); c.1711C>T (p.L571F); c.1110‐2A>G (p.?); c.1859C>G (p.T620S); c.1825A>G (p.R609G); c.1907C>T (p.S636F); g.76304T>G (p.C712G); c.1294G>A (p.V432M); c.2113T>C (p.Y705H); c.109C>T (p.Trp37*); c.1971C>G (p.Y657C); c.1294G>T (p.V432L); c.*1671C>T (p.=); c.1394C >T (p.S465F); c.115G>A (p.E39K); c.2003C>T (p.T668M); c.1027G>T (p.V343F).

### 4.1 Candidiasis

Chronic cutaneous and mucosal candidiasis (CMC) has been identified as a characteristic clinical manifestation in patients with STAT3 dysfunction, including those with dominant‐negative mutations associated with hyper‐IgE syndrome [129, 130]. Superficial *candidal* infections are a recurring phenomenon among patient populations. The most common manifestations of these infections include, but are not limited to, those affecting the oral mucosa and nails. The most frequently isolated species in this regard is *C. albicans* [131, 132]. This susceptibility is closely associated with impaired IL‐17‐mediated immune responses resulting from Th17 cell dysfunction [133]. Research indicates that mice lacking the IL‐17 gene exhibit broad susceptibility to mucosal fungal infections such as *C. albicans* [134]. It is noteworthy that in human subjects, loss of IL‐17 pathway function due to defects in the STAT3 gene is manifested in a distinct manner. Patients exhibit a more limited spectrum of susceptibility, with existing evidence indicating that they primarily develop CMC [134]. CMC is a relatively common fungal infection in humans [135]. *Candida albicans* is a common commensal of the skin and gastrointestinal tract in healthy individuals, but it can become an opportunistic pathogen in individuals with severe T‐cell impairment, whether qualitative, quantitative, inherited, or acquired. This can result in CMC, which often presents as severe recurrent oral thrush [134]. A representative clinical condition is STAT3‐HIES, a form of hyper‐IgE syndrome caused by dominant‐negative (DN) mutations in the STAT3 gene. This syndrome is now widely recognised as a primary immunodeficiency disorder, one of whose hallmarks is increased susceptibility to fungal infections. Most patients with STAT3‐HIES harbour STAT3 LOF mutations that impair the development of IL‐17‐producing T cells. This immunodeficient state underlies their susceptibility to CMC, which presents as recurrent or persistent infections of the skin, nails and mucous membranes by *C. albicans* [52, 136]. Deficits in IL‐17 production and Th17 differentiation led to a markedly weakened immune response against *C. albicans* at mucosal and skin barriers, which results in a failure of local antifungal defence [83, 137].

Evidence from both IL‐17A‐deficient mouse models, as reported by Saijo et al. and from patients with hyper‐IgE syndrome exhibiting defective IL‐17 signalling converges to underscore the essential role of IL‐17 in antifungal immunity. Both groups demonstrate increased susceptibility to systemic *C. albicans* infection [138]. In patients with oral candidiasis, transcriptomic analysis using Ingenuity Pathway Analysis identified STAT3 as the central upstream regulator within a network involving IL‐22 and IL‐17RA. IL‐17 and IL‐22 signal through distinct downstream pathways but act cooperatively to clear *Candida* infections in the oral mucosa [20, 139].

### 4.2 Aspergillosis

Patients with STAT3 mutations are at increased risk of developing chronic pulmonary *aspergillosis* [140]. In response to *Aspergillus fumigatus* infection, macrophages and neutrophils initially recognise the pathogen. Dendritic cells subsequently process antigens and present them to naïve CD4^+^ T cells, triggering STAT3 phosphorylation and promoting Th17‐mediated IL‐17 production. IL‐17 enhances recruitment of inflammatory cells, particularly neutrophils, to the site of infection and facilitates effective pathogen clearance [14].

A study included 12 patients with genetically confirmed STAT3‐LOF mutations, aged 20 to 55 years, of whom 67% were male. Using healthy controls and patients with other forms of immunodeficiency as comparison groups, the researchers systematically analysed the immune response characteristics of PBMCs following stimulation with *A. fumigatus* antigens [24]. The results showed that neutrophil and monocyte counts were within the normal range in all patients. Immunophenotyping revealed reduced T‐cell counts in one patient, decreased memory B‐cell counts in seven patients, and diminished NK cell counts in seven patients [24, 115]. Patients with STAT3 deficiency showed impaired adaptive immunity, especially reduced IFN‐γ, IL‐17 and IL‐22 production. Th17 function was severely compromised, with nearly undetectable IL‐17A levels. No defects in innate immune functions like phagocytosis or cytotoxicity were found. Pulmonary T cells during infection mainly exhibited a Th17 phenotype, suggesting that Th17 impairment explains increased susceptibility to *Aspergillus* in STAT3‐deficient individuals [141, 142].

Patients with STAT3 deficiency and aspergillosis showed reduced IFN‐γ levels compared to those without aspergillosis [143]. Their CD4^+^ T cells had a significantly impaired ability to produce IFN‐γ when stimulated with *A. fumigatus* antigens [138, 144]. ELISA and FACS results consistently confirmed the diminished IFN‐γ and Th17 responses in these patients [24, 144, 145]. Lung cavities and defects in adaptive immunity, antimicrobial peptides and chemokine production may contribute to pulmonary aspergillosis in STAT3 deficiency [144, 145]. Dual suppression of IFN‐γ and IL‐17 was observed in all Aspergillus‐infected patients, suggesting IFN‐γ acts as a critical compensatory defence mechanism against fungal infection [24].

### 4.3 Fusarium

*Fusarium* is a ubiquitous mould whose potential to infect humans depends on the host's innate and cellular immune status. In patients with STAT3‐HIES, the development of *Fusarium* dermatitis may be linked to structural abnormalities and impaired cutaneous immune barrier function. Impaired IL‐17 production and Th17 differentiation compromise the skin's defence against fungal invasion and alter the skin microbiome, further disrupting mucosal immune defence. Salam Abbara et al. reported four patients with STAT3 gene deletion mutations and cutaneous *Fusarium* infections. The patients, aged 4 to 33 years, had chronic skin lesions starting in the extremities. Biopsies showed deep inflammatory infiltrates with fungal hyphae, and *Fusarium* was cultured from samples. Infections remained localised to skin and subcutaneous tissue without systemic spread. Impaired IL‐17 responses in STAT3‐deficient patients may explain susceptibility to localised *Fusarium* infection [83].

Shi et al. reported a 4‐year‐old girl with facial pruritus and verrucous lesions that progressed over 2 years to involve the entire right side of the face, with exudation and scarring. Fungal hyphae were observed in secretions and tissue samples, and *Fusarium* was identified by TEF‐1α gene sequencing. Genetic analysis revealed a heterozygous STAT3 mutation (c.1027G>T, p.V343F) in the patient. Immunological tests showed mostly normal immune parameters except for a slightly reduced NK cell count. The case supports a diagnosis of STAT3‐HIES and suggests that STAT3 mutations may increase susceptibility to *Fusarium* infections via impaired Th17 immunity [128].

### 4.4 Talaromyces Marneffei

*TM* is an opportunistic fungal pathogen. The geographical distribution of *TM* infections is primarily concentrated in Southeast Asia, southern China and northeastern India. Although *TM* infections are rare in the general population, they are relatively common among individuals with HIV/AIDS, cancer, organ transplants, or immunodeficiency [146]. Zhang et al. reported a *TM* infection case with a de novo heterozygous missense mutation (c.1394C>T, p.S465F) in the STAT3 gene [121]. This mutation resided within the DBD of STAT3 and represented the first documented case of a novel STAT3 mutation linked to TM infections in infants and young children [121]. Impaired STAT3 function results in a compromised adaptive immune response to fungal pathogens. Mutations or functional deficiencies in the STAT3 gene disrupt normal immune cell activity, lead to dysregulated production of inflammatory cytokines, and impair the differentiation of T and B lymphocytes. Consequently, these disruptions increase host susceptibility to fungal infections, prolong the duration of infection and contribute to more severe disease outcomes [121].

## 5 The Clinical Significance of STAT3 in Fungal Treatment

### 5.1 Diagnosis

STAT3 gene mutations are closely related to susceptibility to fungal infections and have the potential to be used as a biomarker. Through gene sequencing, it can be determined whether there is a STAT3 mutation, thereby identifying whether it is the genetic cause of primary immunodeficiency‐related fungal infections [147]. For instance, the Chr17:40,500,425 C>T nonsense mutation located in the NTD of the STAT3 protein has been associated with chronic pulmonary aspergillosis [148, 149]. Furthermore, there is evidence from luciferase reporter assays that multiple high‐frequency mutation sites (e.g., E286G, T716M, K348E) within the DBD and SH2 domains of STAT3 have a functional impact. In contrast to LOF mutations, GOF mutations have been observed to result in sustained activation of STAT3 [147]. In terms of differential diagnosis, it should be noted that STAT1‐GOF mutations (e.g., c.1159A>G) may also cause immune dysregulation and are associated with CMC. The distinction between these two categories can be ascertained through targeted gene sequencing [150].

The production of IL‐17 is considered an important mechanism in antifungal immunity. Fungal infections activate the STAT3‐dependent signalling pathway, stimulating Th17 cells to produce IL‐17. Therefore, IL‐17 may be a potential biomarker for fungal infections [36]. Consequently, the primary focus of immunological biomarker studies in STAT3‐deficient patients is on abnormalities in the Th17 signalling pathway. Among these, reduced IL‐17 levels serve as a key diagnostic indicator, directly reflecting impaired STAT3‐mediated Th17 cell function and correlating with an increased risk of invasive *Candida* infections [36, 151, 152, 153]. Furthermore, the phosphorylation levels of STAT3 (e.g., pY705—STAT3) can serve as a quantitative indicator of its signalling pathway activity. When integrated with an assessment of IFN‐γ responsiveness, this aids in determining the functional state of macrophages [154, 155]. In patients diagnosed with STAT3‐HIES, increased expression of SIRP‐α has been found to play a role in reducing the phagocytic function of macrophages. Additionally, the level of SIRP‐α expression may serve as a promising biomarker for monitoring infection progression, offering valuable insights for clinical assessment and patient care [156].

### 5.2 Treatment

From a therapeutic perspective, conventional antifungal treatment regimens demonstrate significant limitations in patients with STAT3 deficiency. For example, in patients with STAT3‐HIES, there are currently no curative or targeted treatment options available [136].

A deeper understanding of STAT3's role in fungal susceptibility offers insights for new antifungal treatments. Targeted treatments addressing STAT3 mutations have emerged due to improved disease understanding. JAK–STAT pathway therapies show promise, especially in Th17/IL‐17 signalling dysfunction from STAT3 deficiency. Ruxolitinib, a JAK inhibitor, improves STAT1 phosphorylation and helps manage refractory candidiasis and autoimmune hemolytic anaemia [157]. Mechanistically, this pharmaceutical agent has been shown to restore Th17 cell function to a certain extent, thereby reducing the proportion of IL‐17‐positive cells from pathological levels back to near‐normal ranges [158]. In animal models, IFN‐γ supplementation has been shown to reverse mucosal barrier dysfunction by modulating abnormal JAK–STAT signalling pathways, thereby reducing susceptibility to fungal infections [159, 160].

Annalisa proposed the ‘trinity’ hypothesis, identifying the IL‐6‐IL‐17‐STAT3 axis as central to autoimmunity and chronic inflammation. IL‐6 induces naïve CD4^+^ T cells to differentiate into Th17 cells through JAK‐STAT3 signalling. Activated STAT3 enhances the production of inflammatory cytokines such as IL‐17 and IL‐21. These cytokines maintain STAT3 activation, forming a positive feedback loop. This cycle progressively worsens autoimmune disease [94]. In light of the aforementioned mechanism, pharmaceutical agents that target the IL—6 receptor (e.g., tocilizumab), IL—17, or STAT3 and are already utilised in the treatment of autoimmune conditions, may also present a novel strategy for enhancing antifungal immunotherapy [56]. In addition, subsequent to undergoing haematopoietic stem cell transplantation in patients diagnosed with STAT3 deficiency, a complete restoration of Th17 cell functionality was observed, thereby indicating that functional correction through immune reconstitution may be a viable therapeutic approach [157].

## 6 Conclusion

In conclusion, the accumulating evidence emphasises a robust association between STAT3 and susceptibility to multiple fungal pathogens. Mutations leading to functional deficiency in STAT3 compromise the immune system's ability to defend against fungal infections, thereby increasing the host's susceptibility. As research progresses, large‐scale, multicenter clinical studies are imperative to further clarify the relationship between STAT3 mutations and fungal susceptibility among diverse populations. Such studies will play a crucial role in establishing more comprehensive diagnostic frameworks and risk stratification models. Moreover, with a more in‐depth understanding of the mechanistic connection between STAT3 and antifungal immunity, therapeutic strategies targeting STAT3‐related pathways present a promising direction for innovation. These approaches have the potential to yield novel interventions. Ultimately, the ongoing investigation into the role of STAT3 in antifungal defence is expected to deepen our mechanistic understanding, providing both a solid theoretical foundation and practical insights for improving the prevention, diagnosis and management of fungal diseases.

## Author Contributions

**Xiaodong Liu:** investigation, writing – original draft. **Fengming Li:** writing – original draft, conceptualization, methodology, investigation, writing – review and editing. **Jing Guo:** writing – original draft, writing – review and editing, conceptualization, methodology. **Jie Wu:** methodology, writing – original draft. **Ningning Dang:** writing – original draft, conceptualization, methodology. **Yuanyuan Li:** writing – original draft, conceptualization, methodology.

## Funding

This work was supported by the China Postdoctoral Science Foundation (2022M710852), the National Natural Science Foundation of China (82102420) and the Natural Science Foundation of Shandong Provincial (ZR2024QH034).

## Consent

All authors have consented to publication. The patient and other participants provided written informed consent for their personal or clinical details along with any identifying images to be published in this study.

## Conflicts of Interest

The authors declare no conflicts of interest.

## Data Availability

The authors have nothing to report.
